# Shifting the Focus: A Photovoice exploration of the benefits and barriers of having a pet while experiencing homelessness

**DOI:** 10.1371/journal.pone.0295588

**Published:** 2024-03-13

**Authors:** Gemina Garland-Lewis, Emily Scott, Vickie Ramirez, Peter Rabinowitz

**Affiliations:** 1 Center for One Health Research, Hans J. Rosling Center for Population Health, Department of Environmental and Occupational Health Sciences, University of Washington, Seattle, WA, United States of America; 2 School of Medicine, University of Washington, Seattle, WA, United States of America; 3 Department of Medicine, University of Colorado, Denver, CO, United States of America; Harvard University HSPH: Harvard University T H Chan School of Public Health, UNITED STATES

## Abstract

While experiencing homelessness with a pet can present unique challenges, it can also provide physical health, mental health, and social benefits. A Photovoice project with adolescents and adults experiencing homelessness with a pet was conducted in Seattle, Washington, USA in 2018–2019 to explore the experience of homelessness with a pet, the impact of the human-animal bond on the health of each, and to drive policy changes to help support people experiencing homelessness (PEH) with pets. Twenty-five people spanning a range of age groups, genders, and living situations were recruited primarily through homeless-services providers, 19 of whom completed the study and created over 900 images. One-on-one semi-structured interviews were conducted with each participant to review printed photos. A key theme emerging from the interviews and photos was the importance and strength of the human-animal bond, providing mental and physical health benefits, and motivation for daily activities or making/maintaining positive changes. Challenges related to homelessness with a pet included barriers to access services, housing, and employment. Participants provided policy recommendations to improve support for PEH with pets. Multiple public exhibitions of images from all participants were held, reaching service providers, policy makers, and the general public. Visitor feedback included statements of positive change in attitudes towards PEH with pets among housed individuals. Collectively, the images, stories and themes deepen our understanding of homelessness with pets, leading to increased empathy and potential for policies that benefit the health of PEH and their pets.

## Introduction

### Homelessness with a pet in the United States and Seattle-King County

Estimates of the burden of homelessness in the United States stem from the annual Point-In-Time Count, when at least 582,462 people were experiencing homelessness in a single night of January 2022 [1]. The Seattle-King County region hosts one of the country’s largest homeless populations with 13,368 people experiencing homelessness (PEH) in 2022 [2]. Although the total number of PEH with a pet in Seattle-King County is unknown, reported pet ownership among unsheltered PEH in Los Angeles County ranged between 9–12% with 48% reporting that they were denied shelter services due to their animal [3]. We estimate the proportion of PEH who own a pet, in Seattle-King County is higher than the national average due to the overall high levels of pet ownership in the area, the presence of organizations devoted to the care of pets belonging to homeless community members, and the relatively accepting local regulations regarding animal access, including into many homelessness service facilities [4, 5]. Despite this more accepting environment, PEH with pets in Seattle-King County report that their animal serves as a barrier to accessing medical services and are more likely to seek care for their animal than for themselves [6]. This manuscript utilizes the term “pets” to be inclusive of companion animals, emotional support animals and service animals.

### The human-animal bond in homelessness

The mutual benefit between humans and pets has been well-established, spanning from improved physical health and safety to companionship and psychosocial support [7]. The importance and strength of the human-animal bond may be amplified during homelessness [8, 9]. PEH and their pets often spend the entirety of the day together as many PEH do not have a safe location for their pets to stay while they work or complete other daily activities. Additionally, PEH face heightened threats to their physical and mental health, allowing for a greater potential magnitude of benefit from the human-animal bond [10, 11]. The social and mental health benefits of the human-animal bond during homelessness have been repeatedly demonstrated [8, 9]. PEH frequently describe their pet as a vital, and at times their sole, source of unconditional love, acceptance, and companionship [12–16]. PEH report their pet provides them with a sense of motivation and responsibility, and protects against high-risk behaviors such as substance use [9, 12, 14–17]. Pet ownership has been shown to be protective against and therapeutic for mental illnesses, such as depression and social isolation, in PEH [12, 15, 18–20]. The physical benefits of pet ownership during homelessness have not been extensively explored but prior studies suggest it provides protection against physical violence, particularly when sleeping in vulnerable areas [12, 19, 21]. The benefits of the human-animal bond during homelessness also extend to the health of the pet as PEH frequently place their pet’s needs above their own [6, 18, 19, 22]. Moreover, recent studies confirmed that cats and dogs belonging to PEH are in good health, comparable to pets belonging to people who are not experiencing housing instability [23–25].

Nevertheless, people and pets experiencing homelessness often face unique or exacerbated health challenges and vulnerabilities. PEH and their pets experience restricted access to basic necessities, and the financial cost of caring for a pet can force PEH to choose between their own and their pet’s needs. Health disparities observed in PEH are caused in part by barriers to accessing medical care that can be exacerbated by pet ownership when medical facilities and service providers restrict pet entrance [8, 11, 26]. Similarly, pet ownership is frequently reported as a barrier to shelter services, employment, and long-term housing [8, 9, 12, 16, 17, 19, 27]. It is not uncommon for PEH with pets to forgo shelter, services, medical care access, or long-term housing in order to remain with their pet [14, 17]. And while the psychological benefits of the human-animal bond during homelessness are clear, loss of a pet during this time can be devastating [12, 14]. Finally, PEH can experience harassment and stigma when society sees them as “unfit” to be a pet owner [8, 18–20].

These challenges highlight the unique support and service needs of PEH with pets that often go unmet and/or are difficult to implement, such as pet friendly shelter services [28]. To address the structural barriers this community faces, Kerman et al. proposed a framework for interventions at the policy and service level, and highlighted the importance of shifting negative public perception and stigma directed towards PEH with pets [26]. However, there has been no research exploring how to implement this “public-level intervention” aimed at altering negative public perceptions towards PEH with pets [26].

### Photovoice

Photovoice is a qualitative participatory action research method which engages participants directly in the research and health promotion process through taking and reflecting on photos to understand and promote dialogue around a community issue and achieve a goal such as public advocacy or health promotion [29–31]. Photovoice strives to build a sustainable and equitable partnership between community members and researchers that empowers participants to define the narrative of their lived experience and identify the needs and strengths of their self or community [29–31]. Social change is an integral part of participatory action research and Photovoice can serve as both an intrinsic health promotion intervention, as well as a community dialogue and policy advocacy tool through elevating the voice of participants and including policy-makers and community leaders throughout the project [30, 32, 33].

Photovoice has been demonstrated to be an effective intervention with PEH that directly empowers and benefits participants, despite the challenges that some participants face in attending Photovoice workshops due to the daily survival demands of homelessness [34, 35]. However, many Photovoice projects, including with PEH, fail to include integral Photovoice components, namely, community engagement and the achievement of action-based goals through policy or community-level change [30, 32, 34]. Thus, many researchers suggest that community or policy change should be included as a research outcome of Photovoice projects, especially related to working with homeless communities [30, 32, 34, 35].

In partnership with a study focusing on health-seeking behavior of PEH with a pet in Seattle [6], we conducted a Photovoice project that aimed to 1) explore the experience of homelessness with a pet and the impact of the human-animal bond on the health of each, 2) elicit empathy for PEH with pets among members of the housed community via a series of public exhibits, and 3) to inform policy changes designed to support PEH with pets. This study was a collaboration between the University of Washington’s School of Public Health, School of Social Work, School of Law, and School of Nursing, and development of exhibit and other advocacy materials was informed by all groups for a multi-faceted approach to the design, reach, and impact of the project.

## Methods

### Participant recruitment and Photovoice training

Recruitment took place from October 2018 through April 2019, in Seattle, Washington via collaboration with service providers/shelter spaces utilized by PEH with a pet (e.g. the One Health Clinic; city-sanctioned encampments), referrals from existing participants (snowball sampling), and, rarely, cold contact when encountering people asking for spare change in public settings with their pet. Two participants had existing relationships with the first author due to previous projects in the community. Due to funding constraints, participation was limited to persons whose primary location was Seattle and who were fluent in the English language, however no one who was interested in the study was turned away due to these criteria. As this was exploratory research with a demographic who often experiences high barriers to follow-up, no targeted enrollment was set. However, recruitment aimed to at least match the range of participants found in Photovoice studies with PEH in our review of existing publications (5–13 participants).

All recruitment and following meetings took place one-on-one instead of in a group setting because of logistical difficulties across the project’s demographic and a desire to recruit a diverse mix of participants. Prior to enrollment, a trauma trigger plan was set in place with project collaborators at the University of Washington’s School of Social Work in case any aspect of participation raised emotions that needed professional counseling. All aspects of this study were reviewed and approved by the University of Washington Institutional Review Board (STUDY00005322), including the use of photos for both data analysis and public exhibition. Due to the nature of this study and its associated exhibit, researchers had access to information that could identify individuals both during and after data collection for contact purposes.

The first author, a female researcher with lived experience in homelessness and experience in both qualitative research and interview methodologies, was responsible for all study visits and interviews. During the first visit, informed consent was obtained orally after the participant was read the study information and consent forms by a study team member and provided space to ask any questions. Participants were provided instruction on Photovoice and received study supplies: a waterproof bag containing two disposable cameras, a waterproof notebook and pen to record information during the project, photo releases for any identifiable people in their images, and study team contact information. In addition, a Tips and Thoughts for Your Photovoice Participation guide designed by the study team was provided S1 File. This document focused on sharing emotions and experiences through photography and highlighted communicating how having a pet has impacted the lived experience of homelessness, as well as ideas/prompts to help focus and inspire images to this extent. Basic photography lessons were also offered at this time, if the participant wished to receive such instruction.

The study team contacted participants to check in one week after the cameras were provided via the information received during the consent process. In addition, participants were able to reach out to the study team at any point with questions or updates via text message or phone calls. Once participants indicated they had finished shooting, a second meeting was arranged to collect cameras to develop film. Prints were made to review with participants and negatives were scanned for the purpose of data analysis and a digital backup.

### Semi-structured interviews

Once prints were received from the photo lab, participants were contacted to set a third meeting to review and discuss the photos they had created, with the meeting place determined by the participant. At this meeting, the interviewer conducted one-on-one, semi-structured interviews in which they responded fluidly to how participants wanted to review and discuss their images, utilizing the Objective, Reflective, Interpretive, Decision (ORID) framework [36]. If these four components of ORID did not naturally occur, follow-up prompts targeting each part of the acronym were included, such as asking for their reflections on the experience, their intentions/hopes for what their images would communicate, and any suggestions for service or policy changes that would be most beneficial to them in their experience with their pet. The ORID approach was preferred over the other common Photovoice discussion methodology, SHOWeD [30, 37], due to its heightened applicability to one-on-one vs group discussions. Sessions were audio-recorded for later transcription and field notes were recorded by the study team throughout the interview. No repeat interviews were conducted. Photo releases were collected and participants were asked to document in what ways they agreed to have the images they made used (internal, educational, public exhibit, online), whether or not there were images they did not want shown in any/all types of use, and how they would like to be credited (or remain anonymous) when any of their images were used. Monetary and non-monetary compensation for time was provided at two points throughout the data collection process: a bag of dog or cat food following the second meeting and $30 following the third meeting. Participants were also given the prints of all of their images at the end of the third meeting. Negatives were held by the study team in a secure, climate-controlled location in case participants requested additional prints.

### Interview transcription and data analysis

Transcriptions from interviews were coded by the first and third authors using ATLAS.ti V.8.4.18.0. Transcripts were not returned to study participants for comment or correction. Deductive and inductive coding strategies were used to identify study themes to be used in parallel with the other branch of the study assessing owner and pet health and barriers to health and housing which has been previously published [6]. As the interviews of both branches of the study addressed similar themes, the authors utilized the same codebook and codes for both sets of interviews S1 Appendix. Deductive codes were selected and defined *a priori* based on themes defined in previous work and other publications on PEH and pet ownership by the first and third author (GGL and VR). As additional themes emerged from the data, inductive codes were added and defined in collaboration by these same authors. Any discordances in coding were discussed and a decision was made to select one code or keep both codes. This resulted in a more reflexive coding practice across the two parts of the study, allowing richer themes to arise. Participant images were coded alongside the interview transcriptions by the first author, utilizing the themes as described above. Additional, Photovoice-specific codes were also created during this process to record various types of data related to photography or to the process, including the frequency with which a certain activity was photographed (e.g. seeking medical/veterinary care, feeding or walking a pet), the intention behind the image (e.g. showcasing a significant item, area, or activity in their life or attempting to communicate a specific aspect of their experience with homelessness), and the style the image was made in (e.g. from their pet’s point of view, documentary, revisiting their past). The guidelines for conducting qualitative studies established by the consolidated criteria for reporting qualitative research (COREQ) and the standards for reporting qualitative research (SRQR) were followed to ensure rigorous standards were upheld [38].

### Photovoice pop-up exhibits

Selection and curation of images for the pop-up empathy exhibits were conducted by the study team with the assistance of a graduate student in communications. Although participants were not directly involved in the final image selection for this show, the study team incorporated notes from the third meeting that detailed the participant’s favorite photos and any they did not wish to be shown. The exhibit and its contents were designed with an empathy-building approach. Elements such as proximity, storytelling, contemplation and nuance, vulnerability, and other facets that have been found to increase empathy in museum spaces were incorporated throughout the design process [39]. A 30’x10’ exhibit space was designed that could be assembled and disassembled easily to accommodate multiple exhibits over a short period of time. Pop-up exhibit locations were designated with a primary focus on areas with high overlap of housed and unhoused community members, as well as a high level of foot traffic and accessibility. A comment station for visitors included a notebook with the prompts: “What did you think before?” “What do you think now?” “What did you respond to?” “Who did you connect with? Why?” and “What would you like to see next?”. The exhibit was displayed at four outdoor locations around Seattle, Washington over the course of 10 days and was shown for six hours at each site. All necessary permits were received from the Seattle Department of Parks and Recreation, the Downtown Seattle Association, and the University of Washington.

Additionally, the exhibit was on display from January-April 2020 at the School of Law gallery and March–September 2022 at the School of Social Work gallery at the University of Washington, representing the departments in which the partner investigators for this project are based. Visitors could voluntarily provide comments on the exhibit via a visitor journal (pop-ups) or comment boards (Schools of Law and Social Work). Additional, future exhibits are planned at the time of this publication.

## Results

Project results include those focused around the process of Photovoice (“Photovoice Participation and Reflection”), content emerging from the study (“Themes Found in Photography and Interviews”), messaging desires from participants (“Intended Communication”), and actionable outcomes resulting from the above (“Policy and Service Provision Recommendations”; “Empathy Exhibit”).

### Participants and demographics

Twenty-five persons were consented into the study and 19 (76%) completed all aspects. Of the six that did not complete the study, two were unable to be contacted following consent and four reported not having the time to participate. Although specific age was not requested, participants were considered an “adult” (N = 12) or “youth/young adult” (N = 7) based on the types of social services they were accessing (i.e., youth or adult shelters). Self-reported gender pronouns were he/him (N = 9), she/her (N = 8) and they/them (N = 2). Various forms of shelter were represented among the participants, including those in city-sanctioned encampments (N = 5), recently housed (<1 year from enrollment; N = 4), longer-term temporary shelter (N = 3), completely unsheltered (N = 3), work-for-housing programs (N = 2), and vehicles (N = 2).

### Photovoice participation and reflection

Participants used their cameras for a median of 31 days (Interquartile range 27–35) before returning them to the study team. Sixteen percent of participants submitted only one camera of the two cameras provided, and only two participants returned the optional notebooks. Overall, 933 images were created during the study period, 787 of which were high enough quality to be used for analysis. The 146 images excluded from analysis were improperly exposed to the point that no discernable content could be seen. Semi-structured interviews lasted an average of 63 minutes (range: 33–109 minutes). These conversations were held with participants while reviewing their prints to help elicit an understanding of the intention of the photographer and called attention to any inherent viewer biases.

**Fig 1:**
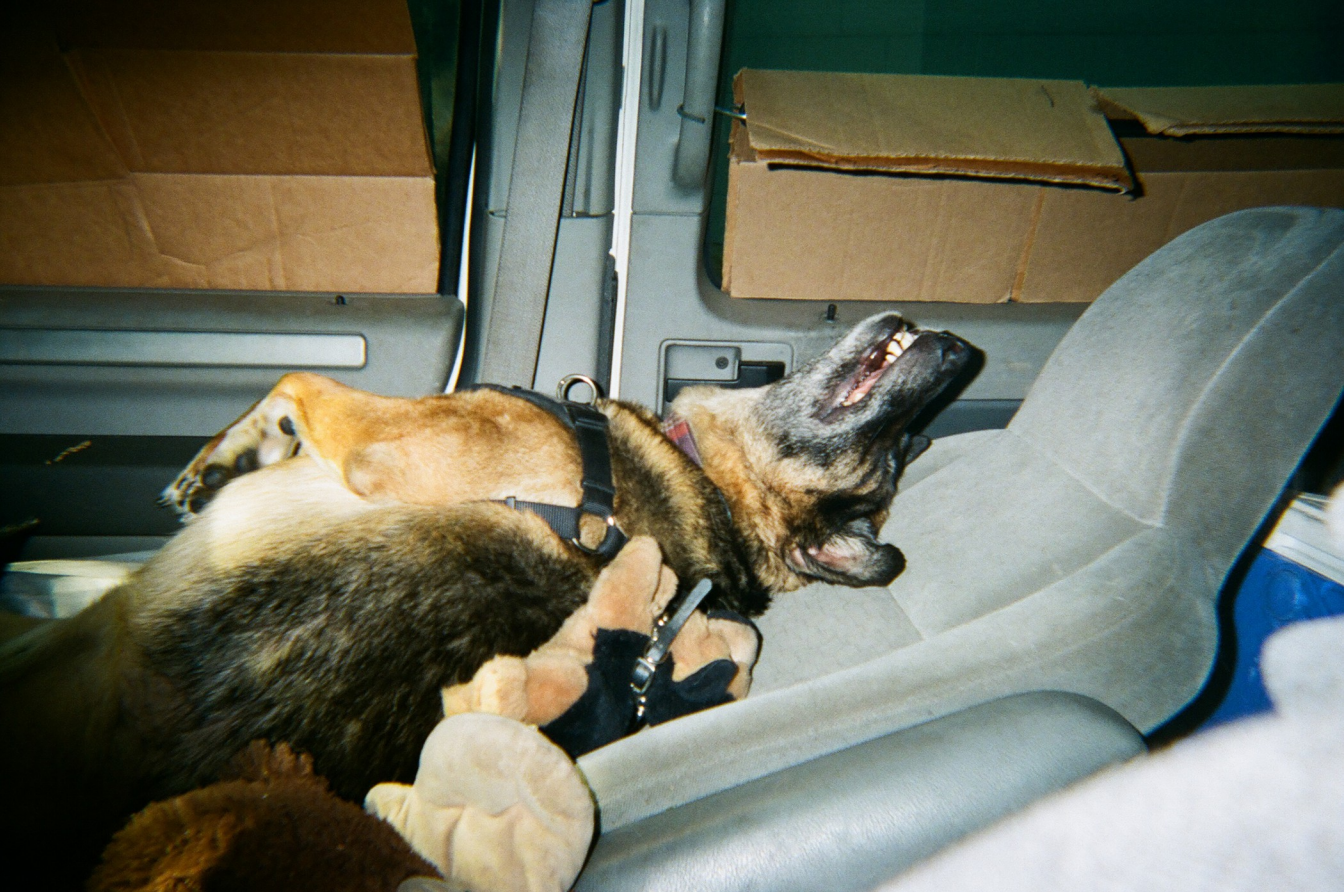
George plays with her monkey after being returned from animal control. “Well you can see she’s kinda the center of my whole world here. Everything. I mean when she was gone, oh my god. I didn’t wanna take any pictures, I didn’t wanna do anything, I wanted my dog back. That was the only thing on my mind. I didn’t deal with the RV for that entire time. I couldn’t. I went up [to the impound lot] and [was given an hour], get what you need for your meds and stuff and let’s go. I didn’t even get clean underwear for myself that day, I grabbed all of George’s stuff to make sure that I got my dog back, because I was going to fight them to the nail and sue the city if I had to [in order] to get my dog back, there was no way they were keeping her from me.” ~ Anonymous and George. Photo used with permission of Participant PV20.

The majority of participants (89.5%) photographed what their life currently looked like, recording a variety of places, people, or activities throughout their Photovoice participation. One participant entered active crisis during the project after her dog was taken by animal control, which created a journalistic, storytelling approach to losing her pet and then having it returned (Fig 1). The remaining participants chose to focus extensively on revisiting places they used to spend time with their pet (sleeping spots, shelters, routines, significant moments, etc.). Overall, some participants staged photos for impact or story while others took a more documentary approach. Although we did not utilize the trauma trigger plan in the course of interviews as no study participant needed that support, we did work with our trauma team to provide support and guidance for the above-mentioned participant during an active pet-related crisis that was unrelated to study participation but occurring at the same time.

All 19 participants offered reflections on their experience during the project. Common positive remarks focused on enjoying being creative and spending time with their animal during the Photovoice process. Several participants noted that they felt the Photovoice project provided a new perspective on their relationship with their pet or their experience of homelessness that they were thankful for. Participants reported they were happy to have the prints, to be able to share them with others, or that the photos brought them comfort. Others reported that the Photovoice experience was beneficial in staying focused and in preparing for daily needs/activities. Two participants described the impact of seeing their lives in print as providing motivation towards finding housing or being more active, demonstrating the ability of Photovoice to intrinsically benefit participants experiencing homelessness.

*I don’t see myself like this because I can never see myself sitting*, *because I’m seeing [it] with my own eyes*, *but I want to capture it like I’m another person*, *you know*? *And look at it and be like ‘Wow*, *what’s going on with the world*?*’ People just pass by seeing us sit all the time*. *Nobody actually asks*, *‘Hey do you need help*?*’*–PV8 (she/her, youth).

*Thinkin’ about the perspective from [my dog’s] point of view…You know I focused more in on that*, *and then sitting back and lookin’ at myself and the situation that I’m in and exactly what is going on*, *and what I need to do and what I don’t need to do even more so*. *It actually sharpened me up a little bit and I’m grateful for that*, *thank you*.–PV13 (he/him, adult).

The most commonly reported difficulty was forgetting the camera. Several people also noted preference for a different visual medium (i.e. digital stills or video). A small number of participants reported performance anxiety or feelings of failure, worried that they would not be able to provide something good enough for the project, either because they could not see the photos they were taking right away or because they did not feel their lives were interesting enough. Both participants who elected to do a retrospective of their experience with homelessness reported feelings of sorrow while retracing their steps, but not severely enough to access the support services provided.

### Themes found in photography and interviews

#### Photography

The two most common themes that arose in images (% of total images showcasing this theme; % of participants who included a photograph of this theme) included the human-animal bond (14.9%; 89.5%) and barriers (both facing and overcoming; 9.9%; 57.9%). Common subjects included photographing their pet with friends (7.6%; 47.4%), daily routine such as walking (11.3%; 63.2%) or feeding their pet (7.1%; 63.2%), and accessing services such as meals, case managers, or medical/veterinary care (3.7%; 4.7%). All participants included at least one image of their living/sleeping space. An average of 84% of a participant’s images contained their pet (range 56–100%). Only two (11%) participants included images of themselves that did not include their pet. Four (21%) participants made a point to take some of their photos from their pet’s point of view, and three (16%) included images of their pet with them at work. The most frequently ascribed code to images and to participant interviews was “chillin’,” a commonly-used slang to indicate a time of relaxation. While this was not included in a particular theme that emerged in the analysis, it is indicative of the times that participants felt most able to or interested in using their cameras.

#### Semi-structured interviews

The human-animal bond was the primary theme arising from interviews. Aspects of this bond were described by all participants while reviewing their photos, six (32%) of whom noted it as their primary driver behind what they wanted to communicate through their images. Common descriptors of pets included “loyal,” “protector,” “my rock,” and “my child.” A reciprocal relationship was often described, focused around statements resembling “we take care of each other.” Both mental health and physical health benefits of having a pet were reported, consistent with non-Photovoice studies of PEH with pets [12, 15, 18–20]. Most commonly (N = 8), mental health impacts were described in reference to overall mood improvement, both through the pet directly giving them joy but also indirectly through seeing that their pet made others around them happy, or in relation to the pet’s assistance with PTSD, depression, and anxiety (N = 8).

*My depression kicked in real bad when I was living in that tent*. *I didn’t come out very much*. *But I knew that because I had him and*, *you know*, *he’d always wake me up in the mornings with a slobbering face*. *I knew I had to go take him out potty and stuff like that*. *I knew when we came back in he’d be right there by my side*. *Lettin’ me know that he was there*. *Him with his personality and him being around*, *it helps me with my depression*.–PV23 (he/him, adult).

**Fig 2:**
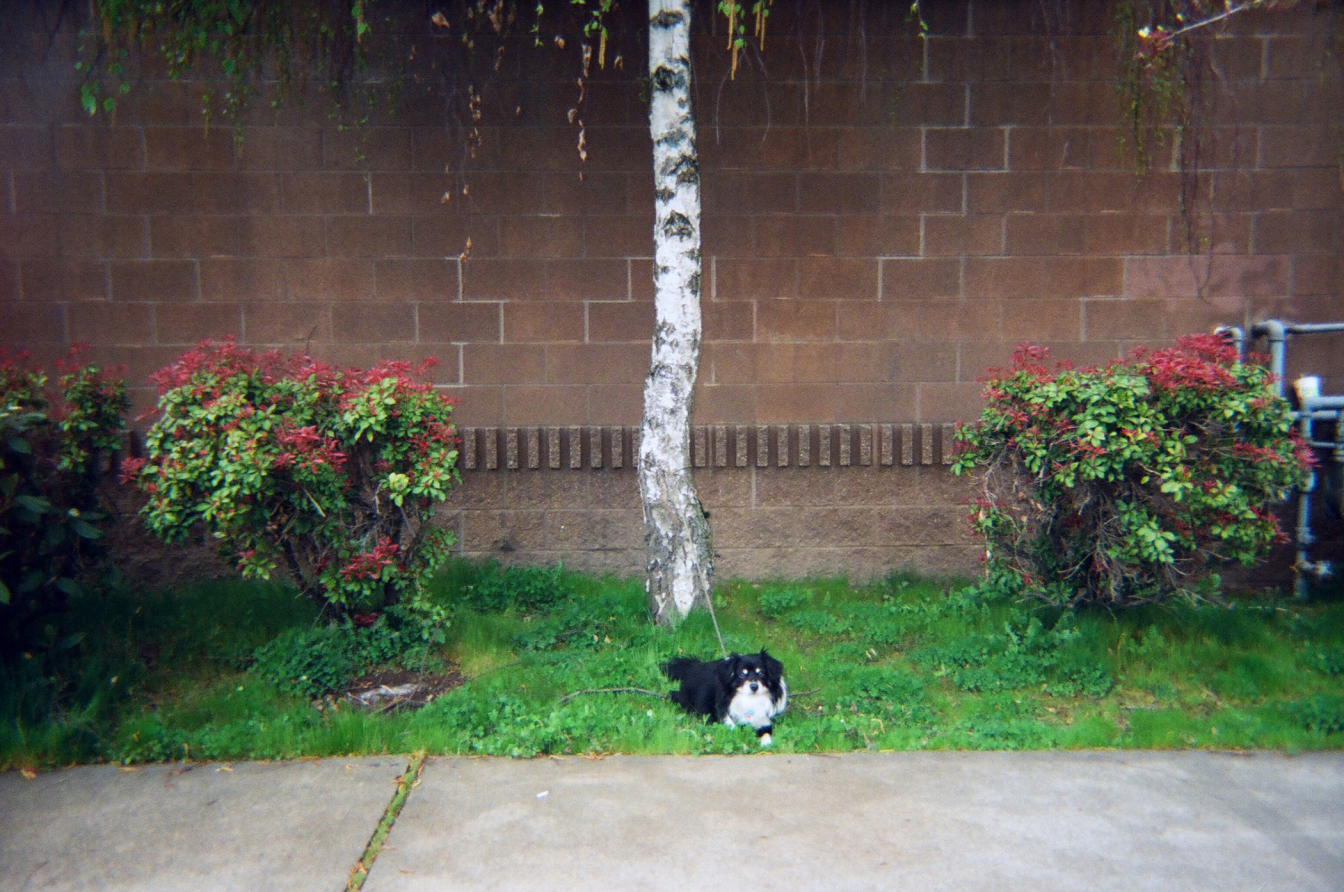
Revisiting dark moments. “This right here, I tried to kill myself right behind this tree. Twice. Two different days in July. And then once in Capitol Hill. He wasn’t with me. He was at my friend’s house. All three times. Cuz I’m like hmmm, then what’s gonna happen to the dog?” ~ Anonymous and Dog. Photo used with permission of Participant PV19.

Mental health impacts were also reported in response in crisis situations (e.g. dog was trained to climb in lap during a panic attack; N = 2) and the pet’s presence mitigating suicidal ideations or attempts at suicide (N = 2) (Fig 2).

Physical health impacts (N = 3) included increased basic activity, assistance with physical disabilities, mitigating flare-ups of physical ailments, and training to assist in a medical crisis.

*I went to the eye doctor and I have a lazy eye that I was born with*, *I was pretty much born blind*, *couldn’t see*, *so I had triple operations*. *Right now I’m farsighted*, *nearsighted*, *legally blind and I have cataracts on one of my eyes now*. *But I have the best eyesight of anybody on the streets*. *My dog is always two feet near me*.–PV13 (he/him, adult)

**Fig 3:**
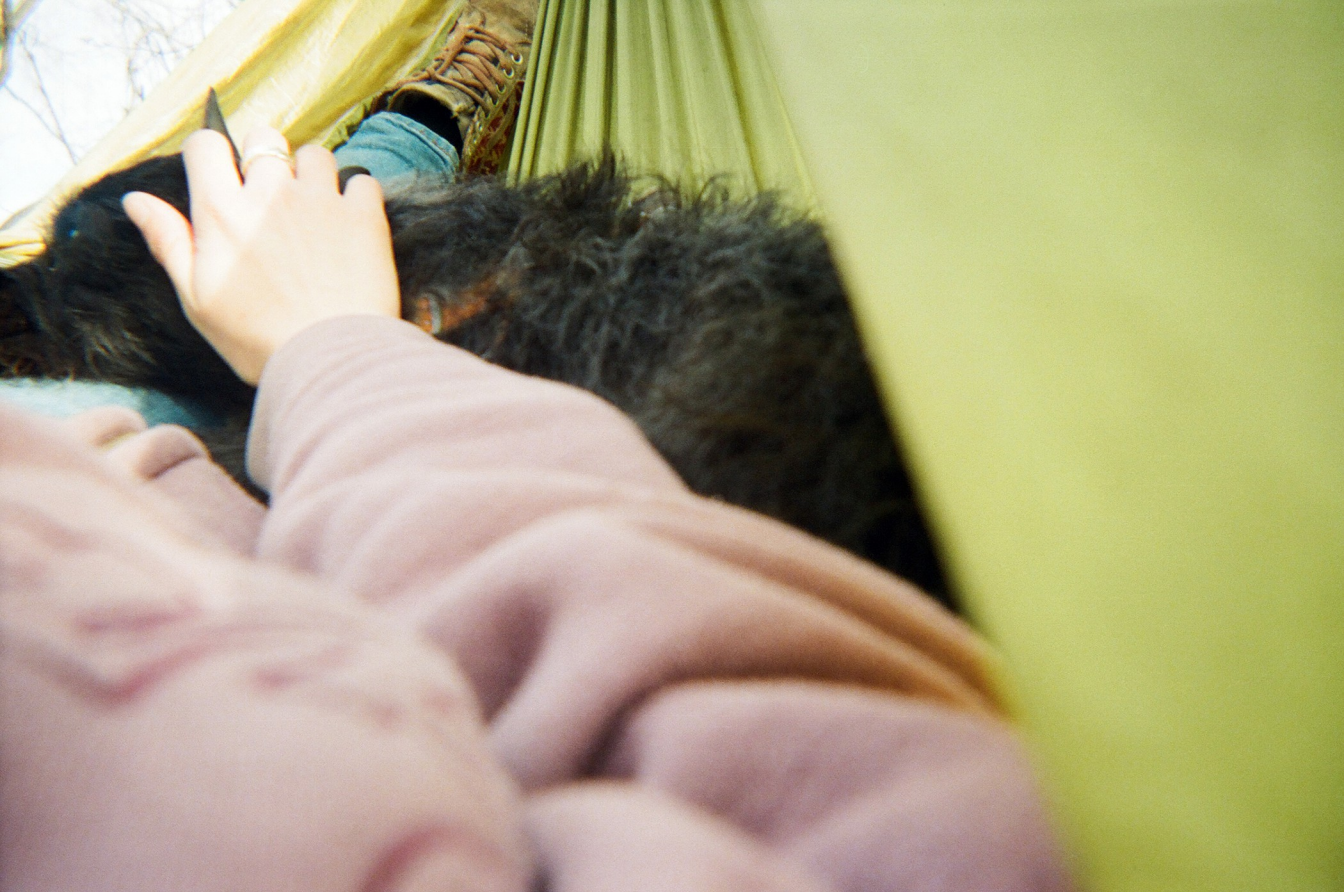
The human-animal bond as motivation. “It’s been substantially comforting to have that bond, that connection with him and it’s helped us strive for a better life for not just him, but ourselves. I don’t think we would have been pushing ourselves to get into an actually housed place before we got Nugget as much as we did. The amount of care and effort it takes to have a being like that in your life makes you have to care about yourself and [it] puts a lot of insight into who you are, too. Cuz you’re teaching this creature to be who he is.” ~ Grace and Nugget. Photo used with permission of Grace Stroklund.

A common role described for pets was that of motivator, with wide-ranging impacts. Two participants noted that their relationship with their dog motivated them to stay sober, while three described their pet as the primary motivation behind finding housing. Four participants independently described their pet as their reason for getting up in the morning, and another four independently noted that caring for their pet had made them take better care of themselves (Fig 3).

One participant felt the opposite and described that they had become so consumed with making sure their dog was well-cared for that they forgot they also needed to provide even basic care to themselves. More commonly (N = 10), participants described ensuring that their pet had food before they had food for themselves, while one noted they had gone without feeding themselves in order to save for veterinary care. Importantly for policy and accessibility implications, five participants described that keeping their pet was a higher priority than housing or work, insofar as that they would not accept either if it came with the condition they had to get rid of their pet.

### Intended communication

Participants described a variety of messages that they hoped their images would communicate to viewers, including both the benefits and difficulties of having a pet while experiencing homelessness, as well as more systemic issues surrounding homelessness and the hardships of going through housing instability.

*I just want people to know stop treating homeless people like they’re lesser than people*. *Because like we’re just like everybody else*, *we all have feelings*, *we all need somebody*, *and we all need a human connection in our lives*. *Like*, *people telling us to get lost*, *that we don’t deserve to be on this earth*, *and ‘Oh*, *you guys don’t deserve to have anything that makes you happy*, *you guys shouldn’t have animals*, *you guys shouldn’t have anything*.*’ Like*, *it’s society’s fault that a lot of people are becoming homeless in the first place*.–PV8 (she/her, youth).

**Fig 4:**
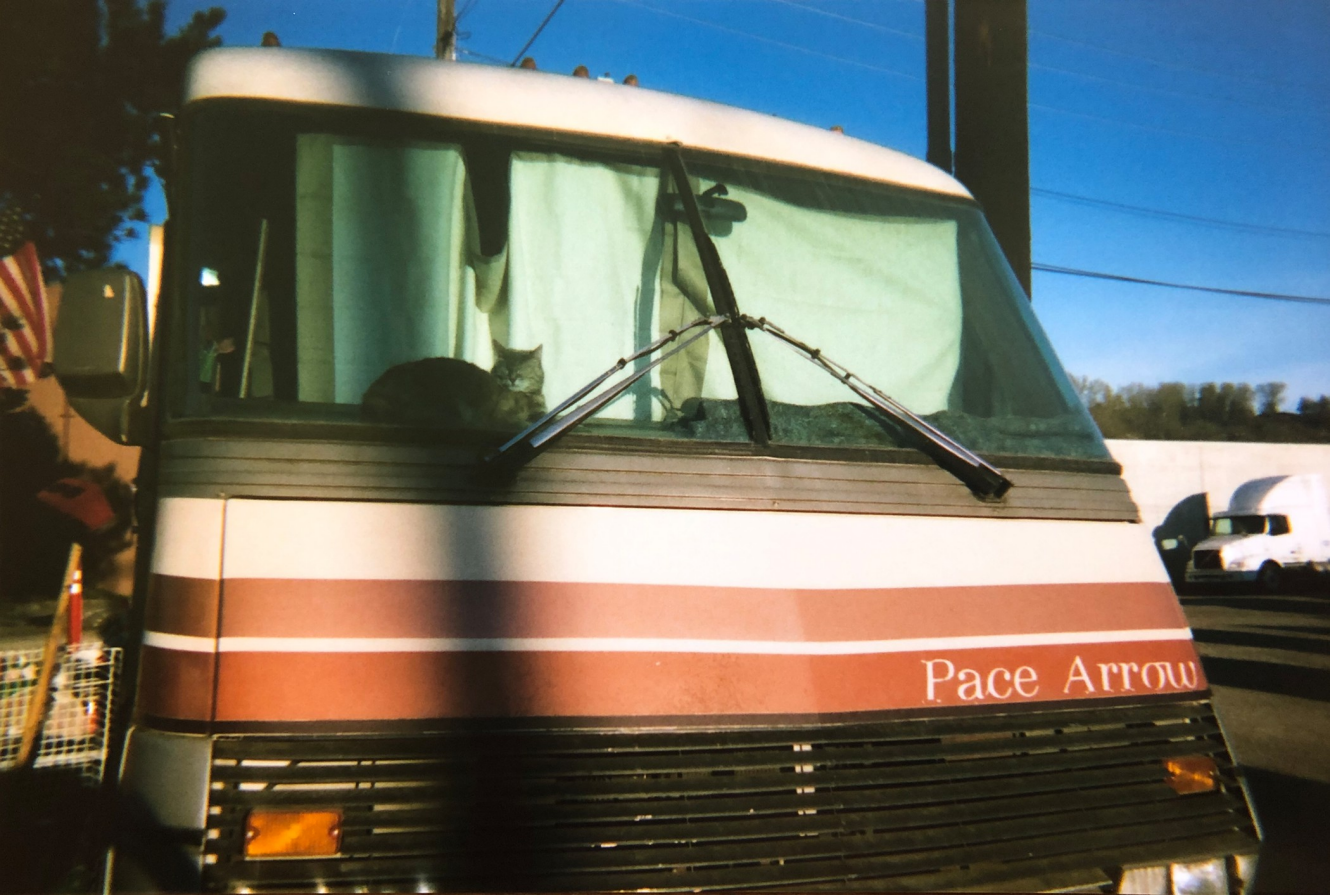
We are all people. “Living homeless with cats in a motorhome is no different than living housed with cats in an apartment or motorhome. We are no different, we just don’t have as stable of an income. You know, we don’t have a $200,000 motorhome, we’ve got a $2,000 motorhome. My point is that life out here has some differences, but we’re all the same. We’re all people, you know? And people have pets. And pets are a huge comfort to these people’s lives. I will eat ramen for the rest of my life before I betray my promise to give them a forever home.” ~ Dee & Walter and Chanel. Photo used with permission of Dee Powers.

The human-animal bond was a frequent theme in participants’ intended message, including the importance of the bond itself—the team or family formed with their pet, the singular importance of the pet in their life, and their devotion to taking care of their pet even above themselves. One participant used her experience of the human-animal bond as a call to the housed community of shared humanity, showing that the comfort that comes from pets was not unique to one’s housing status (Fig 4).

**Fig 5:**
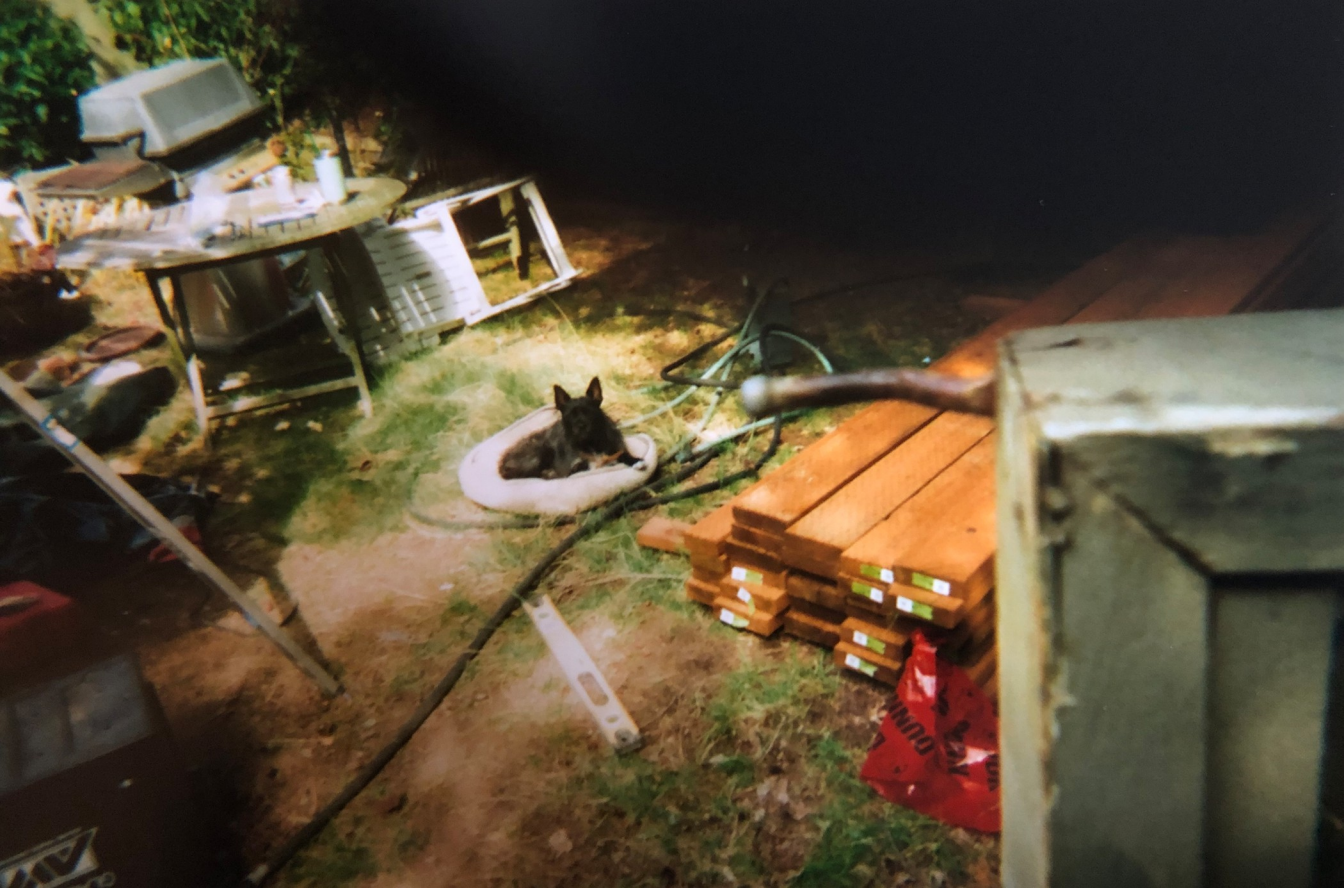
Overcoming a barrier. “[I wanted to communicate] how he incorporated into my work life. Because that was one of the key factors in getting him and being able to keep him and, you know, having him in [my] life if is him being able to come to work with me. If that wasn’t the case then [I] couldn’t have got him.”–David and Dog. Photo used with permission of David Friesner.

Another common theme that arose was the desire to communicate the realities of life as a PEH with a pet. Two participants wanted to show that, even though they did not regret their choices, others should not get a pet while homeless because of the difficulties it would cause in securing housing or work (Fig 5).

Others wanted to call attention to the barriers and difficulties but did not make statements either way about owning a pet.

*I wanted to capture the struggle it was to have a dog on the street*. *Especially if it’s a bigger dog*. *Because there are boundaries*. *I just wanted to show it’s not all fun and games*. *It’s difficult to own a pet on the streets*.–PV18 (he/him, youth).

In contrast, another participant wanted to show through his images that having a pet while homeless was worthwhile, even suggesting starting an organization that specifically provided pets to PEH to help them with their mental health. One participant reflected on the hardships of owning a pet during homelessness, but ultimately wanted to highlight that the benefits outweighed the challenges.

*I knew I was taking on a bigger obstacle in getting [my dog] but I gained so much more than just another obstacle*. *I gained a best friend and he’s also taught me a whole lot*. *And been something stable in my life*. *Been something that I have to make it so I have to remain stable*. *You know I can’t just fuck off and pass out until three in the afternoon and expect [him] to sit there and not go potty*. *You know and not have food*. *So it’s made me stay on my toes*. *I think it’s important to remember to look at what you gained not just what obstacles are going to be in your way when you’re thinking about animals*. -PV6 (he/him, youth).

### Policy and service provision recommendations

Fourteen of the participants provided feedback during photo reviews that was classified as a suggestion for policy changes or service provision that would benefit them in their experience of homelessness with a pet (Table 1). Some of these suggestions directly tied in with images they had taken while others came up in conversation but were not represented visually in their photos. Affordable doggie day care and longer-term foster care were the most commonly suggested service provider ideas (26.3%), the former addressing barriers to finding or keeping employment and the latter addressing crisis situations such as hospitalization, incarceration, or rehab. The most common policy-related suggestion advocated for greater education around service animal regulations (15.8%). Improved accessibility to public transport and grocery stores for people with pets were also suggested.

*When I inform [people] that she is a service dog*, *[they say] “Well you don’t have anything on your dog saying it’s a service dog*, *how am I supposed to know*?*” Well*, *I understand that*, *but [they] get upset with me*, *and at this point I feel like [they] are harassing me because I am trying to explain it*. *Yet [they] want to interrupt me when I am trying to explain that I don’t have to put anything on my dog to display to the entire population that she is a service dog*, *that’s against my human rights*.–PV16 (he/him, adult).

**Table 1:** Policy and service provision recommendations provided by study participants.

*Category*
• Recommendation
***Service Provision Related***
• Affordable/subsidized doggie daycare and/or longer-term foster care
• Affordable/subsidized emergency veterinary care
• Mobile veterinary care that visits homeless encampments
• Publicly accessible pet food banks
• Expand affordable pet services to areas of King County outside of Seattle
• Raise age limitation for youth homelessness services from 25 to 30 years
• Expand shelter policies to allow pets
• Rehab facilities that allow pets
• Option to get packaged meal outside of service provider buildings that do not admit animals
• Access to storage lockers to store large bags of pet food
• Dog parks at Tiny House Villages
***Policy Related to Homelessness***
• Increase public education surrounding service animal laws
• Expand accessibility policies to allow pets on public transportation and stores (ie. Grocery stores)
• End breed-based pet restrictions in housing
• Increase fines for falsely claiming a pet is a service animal

### Empathy exhibits

**Fig 6:**
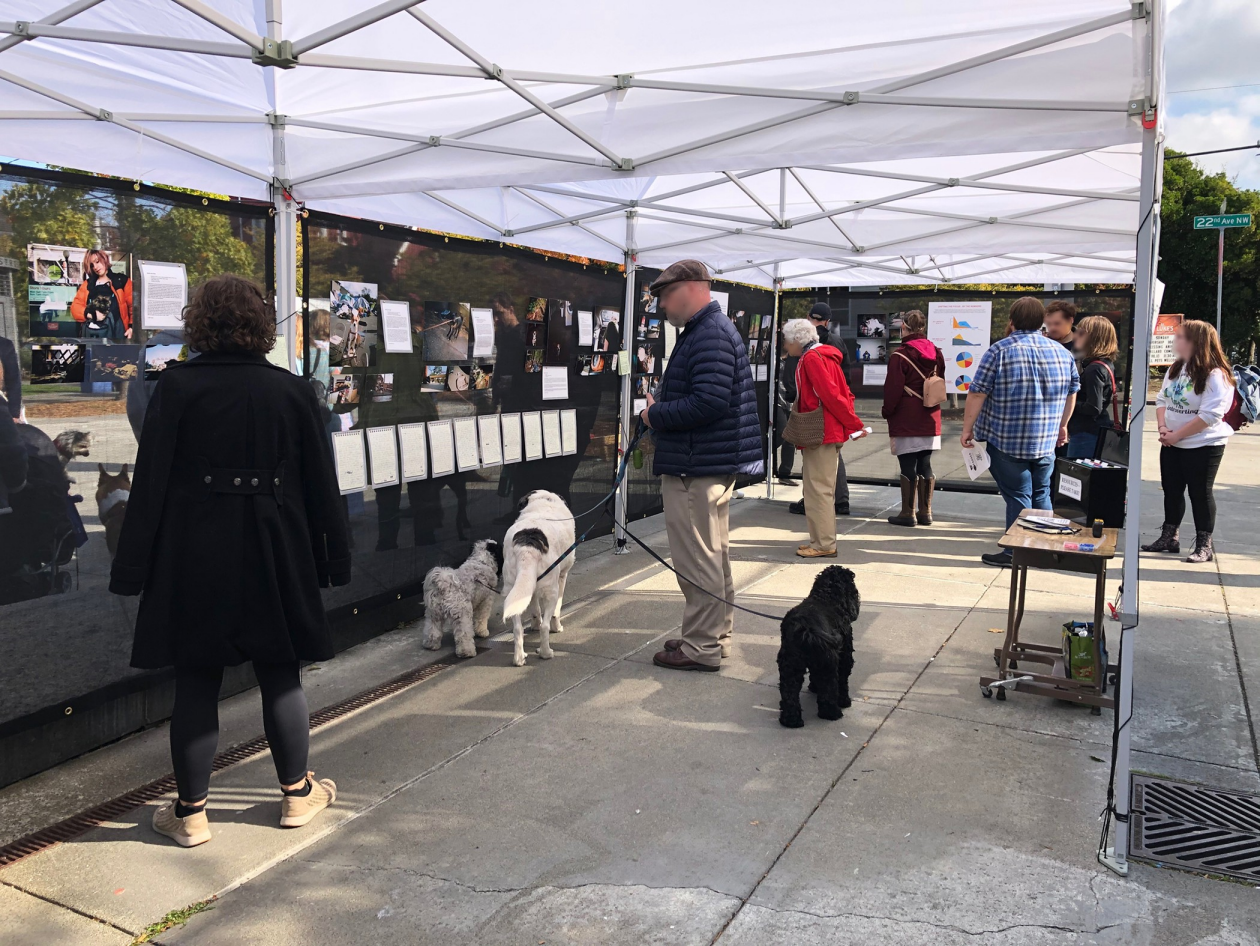
Visitors at the Ballard Commons empathy exhibit. One of four pop-up exhibits of Shifting the Focus around Seattle, which drew over 500 visitors. The 30’x30’ space was set up and dismantled each day and held in strategic public locations with significant overlap between housed and unhoused communities.

Over 500 visitors attended the series of four pop-up exhibits held across a 10-day span in the fall of 2019 (Fig 6). Due to the longer duration, it is unknown how many visitors attended the exhibit when it was shown at the Schools of Law and Social Work. The exhibit concentrated on empathy-building and was specifically designed and located to engage both housed and unhoused members of Seattle. It included 75 printed images representing all 19 participants, text and/or stories associated with the images, two participant notebooks, data visualizations, interactive mapping, and a “Know Your Rights” display on pet ownership in Seattle/King County created by study colleagues at the School of Law based on lessons learned from study participants. Each element of the exhibit was designed to help evoke empathy in one or more ways, for example: storytelling (photos, quotations); proximity (holding/reading original participant notebooks, sharing space with participants or other PEH in attendance); collective journeying (interactive mapping experience across housing statuses); and contemplation and nuance (viewing content, visitor journal prompts). While the exhibit was open to all, specific invitations were sent to those working in service provision and policy surrounding homelessness, as well as study participants–five of whom (26.3%) were ultimately able to attend, engage with other attendees, and provide feedback to the study team. Overall, visitors included study participants, students, academics, local service providers, members of the Seattle Police Department, the unhoused community and the general public. Resource guides for PEH with pets were also available for people to take with them.

Twenty-three comments were recorded at the exhibits. General feedback provided was very positive towards both the design and content of the exhibit and to the participants who shared their stories. No comments were derogatory but several were discarded from analysis as irrelevant (e.g. “Corgis are the best!”). Of the analyzed responses, five commenters specifically recorded what aspect of the exhibit they connected to the most (notebooks and images were the top two), how the stories resonated with them emotionally overall, or one specific story that reached them. There were two comments that juxtaposed a previously held belief about PEH with pets with a new understanding after seeing the exhibit, indicating that engaging with this kind of exhibit can create awareness in a formerly unsympathetic viewer even in a short amount of time.

*I never understood how/why people who are experiencing homelessness could have pets when they can barely support themselves*. *Turns out that maybe if more emotional support animals exist*, *unstably housed people could better their mental health*.–Exhibit visitor.

## Discussion

### The human-animal bond during homelessness and its impact on health

Our Photovoice study with PEH and their pets explored the experience and health impacts of homelessness with a pet in adolescents, young adults, and adults from across a spectrum of current homelessness or housing situations. Consistent with past studies, our participants described pets as a mediator of health through serving as a motivator for daily self-care, finding housing, and maintaining sobriety [12, 14, 16, 17]. Similarly, we found continued evidence of improved mental health and resiliency related to pet ownership while experiencing homelessness [8, 13, 14, 18, 19]. Further, we found that pets serve as a protector against suicide attempts and a support during mental health crises, a potentially life-saving relationship that has been less frequently reported and explored [16]. The physical benefits of pet ownership during homelessness have not been well described outside of physical protection from violence [8, 9]. Our findings described animals (both trained service animals and pets) aiding during physical illness or disability, for example, providing assistance for a visually impaired participant or calming during panic attacks. However, the significant emotional attachment in the human-animal bond had potential negative health effects if combined with restrictive regulations towards pets, as participants reported it would prevent acceptance of services, housing, and employment if it came at the expense of losing the pet.

### Participant-identified service needs and recommendations

Our study also highlighted the unique, often unmet health and service needs of PEH with pets and allowed participants to provide specific service and policy recommendations. In a review of service needs and related interventions for PEH with pets, past studies have focused primarily on pet-friendly shelter/accommodation, free veterinary services and pet food, and other pet-friendly services such as medical services [28]. While our participants mentioned these, the most common service recommendation in our study was access to affordable short-term care or existence of longer-term foster programs, a service that could mitigate the barrier to accessing employment and services that do not permit pets. Multiple of our participants also emphasized the need for public education related to service animal laws and access to training of their pet as a service animal. People with disabilities disproportionately experience chronic homelessness yet face substantial barriers to obtaining or training a service animal [1].

### Empathy exhibits as a means to reduce public stigma

There remain multiple barriers to improving support and services for PEH with pets. Kerman et al. outlined a framework for supporting PEH with pets with interventions at the policy, public and service levels [26]. At the public level, they highlight the need for anti-stigma interventions that change public biases against PEH with pets in order to protect PEH from discrimination associated with pet ownership and to increase public support for services for PEH with pets [26]. We held public exhibits of participants’ photos and narratives aiming to build empathy in the general public and local decision-makers towards PEH with a pet in order to facilitate policies that benefit the health of this community. We collected evidence that these exhibits resonated with viewers, and in some cases, helped facilitate positive changes in individual perceptions and attitudes towards people and pets experiencing homelessness. Moreover, the Photovoice process and the public exhibits allowed participants to contribute ideas to service providers and policy makers that directly impact their health. In addition to the benefits gained from a research perspective, we feel it is of the utmost importance that visuals created by PEH are integrated into the dialogue surrounding homelessness. This is especially relevant when it comes to the topic of pet ownership among PEH, which is often met with criticism from the housed community (“If they cannot take care of themselves, they cannot take care of an animal”) [19, 20, 25, 40]. The opportunity afforded by the empathy exhibits to spotlight visuals taken by people sharing their own experience of homelessness and of caretaking a pet during this time helps to fill a wide gap in such disparate narratives placed on PEH from those without lived experience. This was heightened by the fact that each participant took a different approach to creating their images and was given time to explore the approach they chose, allowing us to share a myriad of perspectives and experiences among PEH.

### Photovoice provides unique insights and impacts for PEH with pets

Photovoice and other similar visual methodologies within participatory action research can illuminate aspects of life for PEH that are not readily captured through traditional research methodology or survey instruments. This study is the first to utilize Photovoice to understand the human-pet joint experience of homelessness. While we confirmed many findings from traditional research methodology regarding the benefits and challenges of experiencing homelessness with a pet [6, 8, 9], the images created by the study participants provided insight into important personal moments with their pets, movements throughout the city, and the formation of community. They also provide additional layers of data via visual descriptors of themes identified during interviews, offering greater detail and opportunity for examination both by participants and the research team. Furthermore, in-depth photo review allowed for participants to offer thoughtful, practical recommendations on service provisions and policy changes to support PEH and their pets. Some visuals additionally confronted implicit biases in the viewer, including even at times the study team. Sitting down with participants to review every photo in-depth was therefore essential to the data analysis process. Finally, participants expressed that they directly benefited through the process of creating and reflecting on their photography, an impact that is often not realized through traditional research methods. Participants reported that Photovoice served as a creative outlet, brought focus in daily life, and provided joy in sharing the importance of their pet. Some participants even reported that the experience served as a motivator to make significant positive changes in their life, such as pursuing long-term housing.

### Strengths and limitations

In addition to the unique contributions of Photovoice as a methodology for understanding the experiences of PEH, several attributes contributed to the strength of this study. First, the study represented a relatively large group for a Photovoice project working with PEH and a diverse representation of perspectives from individuals [34]. Participants were able to keep their cameras for a prolonged length of time, providing opportunity for more breadth, depth, and diversity in their images, as well as time to feel more comfortable with the cameras and the project. Additionally, the empathy exhibits provided an avenue to engage community and policy makers in the discussion and to highlight the work and message of participant photographers. Collaboration across multiple schools at the University of Washington also provided valuable input throughout the project, including trauma support plans from the School of Social Work and tailor-made resources and information from the School of Law that were created in response to study findings and included in public exhibits alongside participants’ photographs.

Past criticisms from participants of Photovoice studies with PEH include researchers not spending enough time up front explaining that the emphasis was on them sharing what they think and feel, not on improving their photography skills [41]. While we did not exclude photo education from this project, we approached this aspect with participant-led methodology in which photography training was offered to everyone should they want it but was not the emphasis of the project. Point-and-shoot film cameras may have limited the extent to which people experimented with photo techniques but also provided a platform through which greater intentionality was exhibited with each image, due to the limited number of photos per roll and the inability to delete frames.

Due primarily to logistical barriers but also an interest in diversifying visual narratives, we opted for a more intense one-on-one experience that could span a greater variety of ages, genders, geographies, sleeping situations, etc. rather than group discussions in the analysis of photos. This is a limitation of this study when compared to other Photovoice projects with PEH as we lacked the benefits that are experienced through support and connection during the group process [35, 41]. It also means we were unable to take an intensive look at any particular group within the participants (e.g. youth, unsheltered, etc). However, in some cases we may have been able to uncover richer data through one-on-one discussions, given that a challenge mentioned by a prior study [41] included participants’ unwillingness to open-up during group discussions of photos. Despite not having a suitable situation for group meetings, ORID procedures were still followed with all participants and participants had the chance to see each other’s work and discuss their experiences at the pop-up galleries. Future Photovoice projects with this demographic could focus on developing the group aspect of this approach while still maintaining a wide array of perspectives.

Another limitation is the staffing issues leading to only the first author coding the images, however we determined that due to this author’s professional work as documentary photographer that they had the most experience on the team reviewing and analyzing images. Future Photovoice studies should include the same analysis methodology and opportunity for reflexivity found in the interview analysis phase. Additionally, we note a limitation in presenting the visual data in this manuscript due to specific journal requirements for a Creative Commons Attribution License (CC BY 4.0) for all photos. Due to inherent difficulties in long-term follow up of PEH, this has limited the photos available for publication in this manuscript.

### Conclusions and next steps

Through imagery, interviews, and exhibits, community members living in homelessness were provided an opportunity to express the health impact of pet ownership while living in homelessness. The results of this were seen beyond the study team, including the general public, service providers, and policy makers who can directly impact participants’ lived experiences with their pets and their health. Response to the exhibit indicates the efficacy of implementing visual media from Photovoice projects to elicit an empathy response among viewers and to help mobilize change within a community. More government agencies and nonprofits are realizing that input from the community they are serving/trying to enact change in is essential (e.g. “Nothing about me without me”). Such insights as provided through this Photovoice project and the empathy exhibit help add to this approach and emphasize the need for and the value of incorporating and elevating the voices, strengths, and policy recommendations of those experiencing the housing crisis firsthand. Expanding this approach may have important implications for policy making at the local level in particular when addressing the health and housing needs of people experiencing homelessness.

## Supporting information

S1 Checklist COREQ (COnsolidated criteria for REporting Qualitative research) checklist. COREQ Checklist filled out by study team to ensure rigorous reporting of qualitive research.
(PDF)

S1 File Tips and Thoughts for Your Photovoice Participation. Written material provided to study participants to help orient them during their Photovoice process.
(PDF)

S1 Appendix Codebook. List of codes and their descriptions used in data analysis, separated by theme and including *a priori* vs *posteriori* designation.
(PDF)

S1 Table Camera usage timeline. Table including the date cameras were given out to participants (date consented) relative to the date they were returned to the study steam.
(PDF)

S2 Table Interview duration. Table including the duration of each participant’s semi-structured interview.
(PDF)

S3 Table Image content by pet. Table including the total number and proportion of each participant’s images that included their pet.
(PDF)
