# Supplementary material for: Shifting the Focus: A Photovoice exploration of the benefits and barriers of having a pet while experiencing homelessness
Source: PLoS One. 2024 Mar 13;19(3):e0295588. doi: 10.1371/journal.pone.0295588 (PMC10936787; doi:10.1371/journal.pone.0295588)
Supplement: S1 File — Written material provided to study participants to help orient them during their Photovoice process. (PDF) [file pone.0295588.s002.pdf]

Tips and Thoughts for Your PhotoVoice Participation:

- While we hope you learn some valuable photography skills, the emphasis of PhotoVoice is on your thoughts, feelings, and lived experience of homelessness with your animal. We want to see your perspective on this part of your life and how it impacts your experience. Because of this, your images will be special no matter what!

- If you are feeling stuck, here are some themes that might help you find the subjects or stories you want to focus on:
    - What are the benefits and barriers of having a pet?
    - Where do you feel welcome/safe? Where do you feel unwelcome/unsafe?
    - What is your daily routine with your animal?
    - Where do access services for yourself and your pet?
    - What is your pet's role in your life?
    - What do you want the world to know about your experience with your animal?

- Remember to always ask anyone who you would like in your picture (and would be recognizable in this photo) if they give you their permission for you to take their photo! Remember to also ask them to sign a consent form for the photos so it can be used in the end of study exhibition or other materials.

- Do not do anything for a photo that puts you, your animal, or anyone else in an unsafe place or position. It's not worth it!

- Use the notepad provided to help write down some thoughts about the pictures you are taking and what you are hoping to explain through them. Taking notes can be a very helpful way to remember what you are thinking, feeling, the weather, time of day, what you were doing, and other useful context for the photograph.

- Keep in mind some of the photography techniques you learned, like the rule of thirds, leading lines, and framing with a foreground, middle ground, and background. Instructions for use of flash can be found on the camera if you need a reminder.

- Your participation is very much appreciated and we are so excited to see the photos you take!

Contact Information:

Gemina Garland-Lewis
gemina@uw.edu
206-317-7677
