# Supplementary material for: Shifting the Focus: A Photovoice exploration of the benefits and barriers of having a pet while experiencing homelessness
Source: PLoS One. 2024 Mar 13;19(3):e0295588. doi: 10.1371/journal.pone.0295588 (PMC10936787; doi:10.1371/journal.pone.0295588)
Supplement: S1 Appendix — List of codes and their descriptions used in data analysis, separated by theme and including a priori vs posteriori designation. (PDF) [file pone.0295588.s003.pdf]

	Code	Description
	**Demographics**	**Code Family: PPT age; Animal species**
a priori	dog	canine
a priori	cat	feline
a priori	youth (25 and under)	25 and under
a priori	adult (26+)	26 +
	**Barriers due to Animals**	**Code Family: Heading including barriers due to animals**
a priori	barrier_work	animals is/is not a barrier to obtaining or going to work
a priori	barrier_transportation	animals is/is not a barrier to getting around town
a priori	barrier_housing	animals is/is not a barrier to getting a home
a priori	barrier_health care	animals is/is not a barrier to accessing health care
a priori	barrier_shelter	animals is/is not a barrier to accessing shelter
a priori	barrier_homeless services	animal is/is not a barrier to accessing services like food or other resources
posteriori	barrier_access building	animals is/is not a barrier to accessing a building, store, etc
posteriori	barriers_none	reporting they haven't had any barrier issues w/animal
	**Health**	**Code Family: Self and animal health**
a priori	general animal health	diet, hygiene, general care, perceived health status
a priori	veterinary care	anything related to veterinary care
a priori	mental health - animal	how their animal pertains to mental health
a priori	mental health - other	mental health not including/unrelated to animal
a priori	drug and alcohol use_self	personal use of drugs and alcohol, inc. as they relate to addiction
posteriori	self health	personal health comments
posteriori	drug and alcohol use_others	impact of others' use of drugs and alcohol, inc. as they relate to addiction
posteriori	hygiene	behaviors pertaining to personal hygiene
	**Available Resources**	**Code Family: Resources in Seattle**
a priori	resources_food_human	where/how human food is obtained
a priori	resources_food_animal	where/how animal food is obtained
a priori	resources_hygiene_human	where/how hygiene (shower, laundry, etc.) obtained
a priori	resources_hygiene_animal	where/how hygiene for pet is obtained
a priori	resouces_healthcare_human	where/how human healthcare is obtained
a priori	resources_vet care	where/how veterinary care is obtained
a priori	resources_shelter	where/how shelter is obtained

a priori	street, live on street	sleeping outside/unsheltered
a priori	non-sanctioned encampment	living in tented encampment not sanctioned by the city of Seattle
a priori	city-sanctioned encampment	living in city-sanctioned encampments (e.g.,Tent City; Nickelsville)
a priori	housing_tent	lives in a tent, whether in community or not
a priori	car,live in a car	live in car or minivan (does not include RV)
a priori	couch surfing/double up	sleeping at a family member's, friend's, acquaintance's house for no longer than 14 days
a priori	emergency shelter	night-by-night, cannot leave personal items
a priori	transitional housing	longer-term shelters
a priori	hotel/motel	hotel/motel
a priori	RV	lives in RV
a priori	housing/apartment	living housed, inc. apartments
posteriori	homelessness_general	broad statements about the experience of homelessness
posteriori	chillin'	hanging out, passing time
posteriori	income_government assistance	eg SSI, disability
posteriori	income_short-term work/alternate income	ways of getting money that don't fall under "employment"
posteriori	income_spanging/flying a sign	spare changing, flying a sign for money, food, etc.
posteriori	resources_case manager	where case management is obtained; other items related to case managers
posteriori	resources_mental health	where/how mental health care is obtained
	**Law & Policy**	**Code Family: All aspects of laws, policies and lawfulness**
a priori	status_companion animal/pet	any comment about animals as pets, issues with access
a priori	status_service animal	any comment about service animals, inc. current status, registry, & issues with access/species
a priori	status_esa	any comment about emotional support animals, inc. current status, registry, & issues with access/species
posteriori	status_esa/sa_aspirational	wants to get an ESA letter or SA status, training, registration
posteriori	prevents illegal activities	owner doesn't break law resulting from knowing they have a pet to care for
posteriori	incorrect information	misinformation, client unaware of actual laws, regulations, etc.
posteriori	jail	any comment about jail, inc. reasons for, time during, & release from incarceration
posteriori	suggestions for city or policy changes	what would improve things for people in this position
	**Harassment**	**Code Family: Harrassment received in the community**
a priori	harrassment housed public	harrassment from housed community

a priori	harrassment police	harrassment, police
posteriori	harrassment bus driver	harrassment, bus driver
posteriori	harrassment shop/restaurant	harrassment, shop/restaurant, business
posteriori	harassment others homeless	harrassment from peers or others in homeless community
	**General Living**	**Code Family: Stories of unhoused life**
posteriori	employment	as contrasted to short-term work or alternate income code above
posteriori	about homelessness or current status	how they lost housing, how long, or current living situation
posteriori	shelter experiences	positive or negative stories about staying at an emergency shelter, inc. with or without animals
posteriori	sweep_tow	experience of having camp swept or RV towed
	**Pet Parenting**	**Code Family: Stories of pet ownership (regardless of housing status)**
a priori	human-animal relationship	relationship, meaning of animal in life
posteriori	animal fights	animal fights
posteriori	play time	dog park, toys, play with other animals, etc.
posteriori	animal sitter	babysitter for animals
posteriori	how acquired animal	how the got their animals (e.g. Craigslist, friend)
posteriori	training/discipline	training/discipline of animal
posteriori	behavior	how animal's behavior impacts access, daily routine, community, etc., inc. food aggression, leash agression, friendliness
posteriori	stealing animals	concern with animals being stolen, actual animals being stolen, attempted stealing
posteriori	protector	how the animal is protective and/or used as protection
posteriori	pet separation	having an animal run away, get taken, and/or the process of getting them back
posteriori	provides purpose	having the animal gives PPT a reason to live, be healthy, be sober, find housing, etc.
posteriori	emotional support not esa	the emotional support an animal brings without it being in the context of their status; animal may or may not be an ESA, this is action-related
posteriori	service animals skills, not service animal	when people talk about skills the animal has even though it is not considered a "service animal"
posteriori	animal_general care	blankets, jackets, brushing, toys, treats, etc.
	**PhotoVoice specific**	**Code Family: For Coding Images**
posteriori	photo_intention_communication	why someone took the photos they did, what inspired them, what they were trying to communicate
posteriori	photo_feeding animal	image shows animal being fed or eating
posteriori	photo_drop in	image was taken at a drop-in space

posteriori	photo_ppt and pet	image shows both PPT and their animal
posteriori	photo_selfie	selfie, may or may not contain animal
posteriori	photo_at work	photo was taken at PPT's workplace
posteriori	photo_wanted to take	description of what or where they would have liked to take photos but didn't
posteriori	photo_reflections	thoughts about the experience or seeing the images
posteriori	photo_in vehicle	photo was taken in RV, car, truck, etc.
posteriori	photo_in housing	photo was taken in PPT's home (if newly housed)
posteriori	photo_revisiting	photo was taken somewhere where they used to sleep, chill, get services, etc.
posteriori	photo_on walk/dog park	on walk or at dog park
posteriori	photo_sleeping spot	for unsheltered PPTs
posteriori	photo_notebook	PPT reading comments from their PV notebook
posteriori	photo_charging	where PPT charges electronics
posteriori	photo_transit	photo was taken on/waiting for public transit
posteriori	photo_asked someone	photo was taken by someone else in order to get person and their animal/s together
posteriori	photo_shelter	photo taken at the shelter they are staying at (can be emergency or transitional)
posteriori	photo_with friend	photo includes a friend/important person not in study
posteriori	photo_significant item	PPT includes something important to them other than animal
posteriori	photo_animal POV	PPT purposefully took shot from animal's point of view
posteriori	photo_documentation	image taken with sole intention to show what life looks like
