# Supplementary material for: Shifting the Focus: A Photovoice exploration of the benefits and barriers of having a pet while experiencing homelessness
Source: PLoS One. 2024 Mar 13;19(3):e0295588. doi: 10.1371/journal.pone.0295588 (PMC10936787; doi:10.1371/journal.pone.0295588)
Supplement: S1 Table — Table including the date cameras were given out to participants (date consented) relative to the date they were returned to the study steam. (PDF) [file pone.0295588.s004.pdf]

Code	Date consented for PV	Date Check in #1	Date cameras returned	# Days cameras out
PV1	10/15/18		12/14/18	61
PV2	11/14/18	Lost to follow up		
PV3	11/21/18	Lost to follow up		
PV4		Lost to follow up		
PV5	12/14/18	Lost to follow up		
PV6	2/25/19	3/4/19	3/26/19	30
PV7	2/27/19	3/6/19	3/7/19	9
PV8	2/27/19	3/6/19	3/7/19	9
PV9	3/1/19	3/5/19	4/22/19	53
PV10	3/3/19	3/10/19	3/29/19	27
PV11	3/3/19	3/10/19	3/29/19	27
PV12	3/10/19	3/17/19	4/27/19	49
PV13	3/15/19	3/22/19	4/11/19	27
PV14	3/17/19	3/24/19	Lost to follow up	
PV15	3/18/19	3/25/19	4/28/19	42
PV16	3/21/19	3/28/19	4/22/19	33
PV17	3/21/19	3/28/19	4/18/18	29
PV18	3/20/19	4/2/19	4/30/19	36
PV19	4/4/19	4/11/19	5/5/19	32
PV20	4/10/19	4/19/19	5/13/19	34
PV21	4/11/19	4/19/19	5/11/19	31
PV22	4/11/19	4/19/19	5/4/19	24
PV23	4/11/19	4/19/19	5/11/19	31
PV24	4/11/19	4/19/19	5/4/19	24
PV25	4/25/19	5/2/19	Lost to follow up	
