# Supplementary material for: Shifting the Focus: A Photovoice exploration of the benefits and barriers of having a pet while experiencing homelessness
Source: PLoS One. 2024 Mar 13;19(3):e0295588. doi: 10.1371/journal.pone.0295588 (PMC10936787; doi:10.1371/journal.pone.0295588)
Supplement: S2 Table — Table including the duration of each participant’s semi-structured interview. (PDF) [file pone.0295588.s005.pdf]

Code	Interview Time (hh:mm:ss)
PV1	00:36:17
PV2	Lost to follow up
PV3	Lost to follow up
PV4	Lost to follow up
PV5	Lost to follow up
PV6	00:49:56
PV7	00:37:40
PV8	00:40:12
PV9	01:44:11
PV10	00:48:22
PV11	00:56:53
PV12	01:36:38
PV13	01:34:08
PV14	Lost to follow up
PV15	01:49:19
PV16	01:16:56
PV17	00:54:30
PV18	00:33:32
PV19	01:10:57
PV20	01:01:04
PV21	00:59:32
PV22	00:41:15
PV23	01:02:03
PV24	00:54:33
PV25	Lost to follow up
