# Supplementary material for: Shifting the Focus: A Photovoice exploration of the benefits and barriers of having a pet while experiencing homelessness
Source: PLoS One. 2024 Mar 13;19(3):e0295588. doi: 10.1371/journal.pone.0295588 (PMC10936787; doi:10.1371/journal.pone.0295588)
Supplement: S3 Table — Table including the total number and proportion of each participant’s images that included their pet. (PDF) [file pone.0295588.s006.pdf]

Code	Images w/Pet	Images w/o Pet	Total Images	Percent w/Pet
PV1	43	1	44	97.73
PV2	Lost to follow up			
PV3	Lost to follow up			
PV4	Lost to follow up			
PV5	Lost to follow up			
PV6	39	11	50	78.00
PV7	33	1	34	97.06
PV8	30	13	43	69.77
PV9	46	5	51	90.20
PV10	22	4	26	84.62
PV11	15	4	19	78.95
PV12	42	0	42	100.00
PV13	49	0	49	100.00
PV14	Lost to follow up			
PV15	41	11	52	78.85
PV16	34	2	36	94.44
PV17	51	2	53	96.23
PV18	16	0	16	100.00
PV19	38	18	56	67.86
PV20	38	17	55	69.09
PV21	29	23	52	55.77
PV22	27	2	29	93.10
PV23	38	0	38	100.00
PV24	29	13	42	69.05
PV25	Lost to follow up			
			787	
